# Harm reduction implementation among HIV service organizations (HSOs) in the U.S. south: a policy context analysis and results from a survey of HSOs

**DOI:** 10.1186/s12913-022-08277-8

**Published:** 2022-07-13

**Authors:** Megan C. Stanton, Samira B. Ali, Katie McCormick

**Affiliations:** 1 Department of Sociology, Anthropology, Criminology and Social Work, Eastern Connecticut State University, 83 Windham St, Willimantic, CT 06226 USA; 2 Graduate College of Social Work, University of Houston, Houston, USA; 3 Steve Hicks School of Social Work, University of Texas at Austin, Austin, USA

**Keywords:** HIV, Harm reduction, Implementation science, Policy

## Abstract

**Background:**

HIV service organizations are integral to serving communities disproportionately impacted by the HIV and opioid epidemics in the U.S. South. Addressing these intersecting epidemics requires implementation of evidence-based approaches, such as harm reduction. However, little is known about the extent to which Southern HIV service organizations implement harm reduction. This manuscript examines: 1) the implementation context of harm reduction in the South, 2) Southern HIV service organization implementation of harm reduction, and 3) the impact of different contexts within the South on HIV service organization implementation of harm reduction.

**Methods:**

To examine implementation context, authors analyzed nation-wide harm reduction policy and drug-related mortality data. To examine HIV service organization implementation of harm reduction, authors performed frequency distributions on survey data (*n* = 207 organizations). Authors then constructed logistic regressions, using state mortality data and policy context as predictors, to determine what contextual factors predicted HIV service organization implementation of harm reduction.

**Results:**

Drug-related mortality data revealed an increased need for harm reduction, and harm reduction policy data revealed an increased political openness to harm reduction. Frequency distributions revealed that approximately half of the HIV service organizations surveyed reported that their organizations reflect a harm reduction orientation, and only 26% reported providing harm reduction services. Despite low utilization rates, HIV service organizations indicated a strong interest in harm reduction. Logistic regressions revealed that while increased mortality rates do not predict HIV service organization implementation of harm reduction, a harm reduction-friendly policy context does.

**Discussion:**

This study highlights how regions within a high-income country can face unique barriers to healthcare and therefore require a unique understanding of implementation context. Study findings indicate a rapidly changing implementation context where increased need meets increased political opportunity to implement harm reduction, however there is a lag in HIV service organization adoption of harm reduction. Financial resources, capacity building, and continued policy advocacy are required for increased HIV service organization adoption of harm reduction.

## Background and significance

The U.S. South (hereafter referred to as ‘the South’) is profoundly impacted by the HIV epidemic. The South accounts for 52% of new HIV diagnoses yet only 38% of the nation’s population [1]. The “Deep South,” which consists of Alabama, Florida, Georgia, Louisiana, Mississippi, North Carolina, South Carolina, Tennessee and Texas, has been particularly impacted by the HIV epidemic, compared to other U.S. regions [1]. HIV in this region has disproportionately impacted groups facing systemic barriers to care, such as Black and Latinx gay and same gender loving men and transgender women [1, 2]. HIV Service Organizations (HSOs), which include AIDS service organizations, community based organizations and federally qualified health centers, are integral to serving communities disproportionately impacted by the HIV epidemic [3–5]. Though still under-resourced compared to other U.S. regions, the South has seen a 31% increase in HIV funding of HSOs in the past 10 years [1] due to public and private investment in Southern-specific initiatives to meet the need for HIV prevention and treatment [6]. Capacity of Southern HSOs has also been foregrounded through the U.S. Department of Health and Human Services “Ending the HIV Epidemic” plans which set ambitious goals, such as reducing the number of new HIV infections by 90% by 2030, and has earmarked money for the highest HIV prevalence counties, more than half which are in the South [7].

This increased investment in HSO capacity has aligned with recent increases in opioid misuse and overdose deaths in this same region and among the same communities disproportionately impacted by HIV [8, 9]. The opioid and HIV epidemics are intertwined in many communities due to similar structural determinant factors, such as economic marginalization and obstructed access to quality healthcare, as well as through the potential for HIV to transmit through intravenous drug use [10]. Addressing these dual epidemics in the South requires implementation of evidence-based approaches which center the role of social determinants of health in their theories of change and provide client-centered, non-stigmatizing, non-coercive services [11–13]. Harm Reduction (HR) is one such approach [14].

HR is a research-supported approach that aims to reduce the negative consequences of health behaviors (i.e., drug use, sex) without necessarily reducing or eliminating them altogether [14, 15]. The principles of HR recognize that certain health behaviors provide benefit to individuals, and thus promotes individual autonomy and choice across a broad spectrum of strategies to reduce accompanying harm [14, 16]. Specific HR services, such as syringe service programs (SSPs) or medication for opioid use disorder (MOUD) reduce the harms associated with drug use and HIV and Hepatitis transmission [17–23]. HR is also an organizational approach to person-centered and community-responsive service provision which improves individual health outcomes, strengthens client-provider relationships and retention in care and transforms organizations [15, 17, 24–27]. Despite evidence of positive health outcomes associated with HR approaches, policies impacting HR- for example policies that support SSPs, aid the distribution of overdose reversal medication (i.e. Naloxone) and protect individuals who call emergency services (i.e. ‘Good Samaritan laws’)- are state-specific and lack clarity [28].

Given the relevance of HR to HIV health promotion, the role of HSOs in serving communities impacted by HIV and opioid use in the South, and the current increased investment in Southern HSO capacity, it follows that HSOs could be a critical site for the adoption and implementation of HR into Southern standards of care. However, the political and social environment relating to HR in the South may pose unique barriers to realizing this opportunity. Implementation science highlights the role of ‘outer factors’ in determining organizations’ ability to implement evidence-based practices [29]. Outer factors refer to the service environment, including influential policies, organizational systems and social dynamics relevant to the intervention (e.g., stigma, social acceptability of the intervention) [29]. For HR, some outer factors that influence implementation include the (il) legality of HR services, the intensity of need for such services (i.e., number of overdose events) and social stigma related to drug use [23, 30–32]. Though researchers have identified general regional trends in the consequences of the HIV and opioid epidemics in the South, there is little systematic understanding of the dynamic social and policy contexts related to HR in the South over the past several years. Additionally, there has been no research, to the authors’ knowledge, surveying if and how Southern HSOs currently implement HR approaches, receive HR training, or are interested in doing so. Nor has there been a consideration of the impact of within-South contextual variations on the implementation of HR at Southern HSOs.

In response, this manuscript aims to: 1) use existing nation-wide HR policy and drug-related mortality data to describe the shifting implementation context relevant to HR in the South, 2) report on analysis of primary survey data to examine the extent to which Southern HSOs implement HR, and 3) examine the potential impact of different political and social contexts within the South on adoption of HR among Southern HSOs. Understanding this landscape will help inform policy, funding, and capacity-building initiatives to support Southern HSO uptake of HR.

We will first present findings related to study aim one in the form of review of the opioid epidemic in the South and an analysis of existing legal and drug-related mortality data. We then present study aims two and three separately, each with their respective methods and results sections. The manuscript concludes with a discussion of findings and implications from all three aims.

## Study aim one findings: the political and social context of the opioid epidemic and HR in the south

### U.S. Opioid epidemic

The opioid crisis in the U.S. has been described as a “triple wave” epidemic, referring to three characteristically different, subsequent, yet overlapping and interconnected phases of the epidemic [33]. The first wave was spurred by an increase in opioid prescriptions in the 1990s. Structural factors which increased risk of misuse in the population, combined with this uptick in prescriptions and changes to opioid technology, resulted in increased opioid pill misuse in the early 2000s [33, 34]. The second wave was characterized by an increase in heroin use around 2007, used by some as a cost-effective means to meet the demands of opioid dependence from the misuse of pills [34]. The emergence of the synthetic opioid fentanyl around 2013 marks the third wave of the opioid epidemic and is characterized by dramatic increases in overdose deaths [33].

Early opioid misuse and overdose deaths were concentrated in states with large rural and suburban White communities, such as Kentucky, Maine, and West Virginia [35]. Black communities did not have the same rates of opioid misuse, in part because Black and Latinx patients were prescribed opioids at half the rates of White patients due to provider bias about potential for misuse and racist notions about the pain tolerance of Black and Latinx patients [36–40]. Racialized narratives about drug use also permeated public perspectives of the opioid epidemic [37, 41]. Whereas earlier eras of drug misuse covered by the media among Black communities was met with aggressive and violent criminal persecution (i.e., crack cocaine use and the War on Drugs), the public discourse associated with the opioid crisis focused on addiction as illness and treatment as the required intervention [37, 40, 42, 43]. Furthermore, when Black and Latinx individuals have sought services related to opioid misuse, they have been less likely to receive high quality treatment due to a range of structural barriers including lack of insurance, lack of community services, provider mistrust and bias, and stigma [44–47]. The third wave of the epidemic hit these communities harder than previous waves: Black and Latinx communities have seen a sharp rise in opioid-related fatal and non-fatal overdoses since 2016, with increases in deaths now outpacing Whites [9, 48]. This history of racism is essential context for understanding Southern HSO implementation of HR, since the HIV epidemic in the South has disproportionately impacted Black and Latinx communities and it is therefore Black and Latinx individuals who are primarily served by these organizations.

### Opioid overdose in the deep south

In terms of impact of the opioid crisis, the nine states of the ‘Deep South’ can be split into the ‘Mountain South’ (NC and TN), which has seen high rates of overdose throughout the epidemic, and the rest of the Deep South (AL, FL, GA, LA, MS, SC, TX), which until recently, had seen overall lower rates of overdose [35]. Between 2012 and 2014, the South continued to have lower rates of heroin-related overdose deaths than other regions but had higher relative increases in these rates [49]. According to data from the CDC, by 2019 the South saw an average 47% increase in drug related deaths over 2014 rates, with five states seeing a more than 50% increase in drug-related deaths (FL, LA, NC, TN, SC) [50].

#### HR implementation context in the deep south

HSO implementation of HR is situated within the broader context of healthcare in the South which influences the experiences of communities disproportionately impacted by HIV and, increasingly, the opioid epidemic. Driven by a decentralized public healthcare infrastructure, the South has invested less in its public healthcare infrastructure compared to other U.S. regions. For example, only 1 out of 7 of states in the US Deep South expanded Medicaid (the largest US public health insurance program), thus retaining restrictive eligibility to qualify for Medicaid and keeping more Black and Latinx people uninsured in these states [51, 52]. Additionally, historically and currently, systemic racism, transphobia, and homophobia uniquely characterize social dynamics in the US South, though they permeate the U.S. on the whole. Black same gender loving/gay/bisexual men in the South report more medical mistrust and perceived racism compared to those living in other US regions [52]. The South practices carceral approaches, such as mass incarceration and consequent stripping of human and voting rights, to intervene with complex issues of health often related to unstable housing, poverty, and violence. Sparked by the federal War on Drugs which introduced mandatory minimum sentencing for drug possessions, the South saw a 127% increase in the prison population from 1990 to 2019, while the US as a whole saw an 86% increase [53]. These carceral approaches toward drug possession resulted in political conservatism toward drug policy and little room for HR to be centered in both policy and practice in the South. In sum, the lack of investment in public healthcare infrastructure and promotion of carceral approaches have created an environment where HR (policies and practices) are difficult to implement.

#### HR policy environment in the deep south

In 2014, well after public recognition of the opioid epidemic and just as fentanyl increased drug poisoning mortality, the policy landscape in the South remained hostile to HR strategies. For example, an analysis of HR policy conducted by Fernández-Viña and colleagues highlights that in 2014 no state in the Deep South had any form of legal protections for SSPs [28]. Only North Carolina, which experienced dramatic increases in opioid overdose deaths during the early emergence of fentanyl, had enacted (in 2013) laws protecting Naloxone distribution or laws protecting individuals from prosecution for calling emergency services related to overdose, referred to as ‘Good Samaritan’ laws [54]. It seems policy responded, if belatedly, to increases in opioid related mortality described above. By 2019, all Southern states had Naloxone distribution laws, and all but Texas had Good Samaritan laws [55]. Five of nine Southern states had laws which explicitly authorized SSPs by 2019. Notably, Alabama, Mississippi, South Carolina, and Texas were the only states in the U.S. to not have these explicit legal protections for SSPs by 2019 [28]. However, providing some legal protections for the distribution of syringes does not ensure unimpeded access to these programs. As of 2019, all Deep South states has some prosecutable drug paraphernalia laws on the books, and only North Carolina and Tennssee had legal protections for people who inject drugs, such as excluding drug residue in used syringes from drug possession laws and providing immunity for people who disclose possession of syringes prior to a police search [28]. This policy context influences and is influenced by the social acceptability of HR in the South. Authors recently found that restrictive funding, anti-drug policies, and stigmatizing provider attitudes contribute to resistance to HR implementation in the South [27].

In sum, the period of 2014-2019 saw increasing need for HR in the Deep South, as well as some shifts indicating increasing, though incomplete, political openness to HR (see Tables 1 and 2). Additionally, HR focused organizations, such as the Southern Harm Reduction Coalition and other local grassroots organizations, were pushing the envelope of HR acceptability during this time through service provision, organizing, and advocacy. The breakthrough successes of these organizations increased the visibility and social and political accessibility of HR in the South [56]. This evolving implementation context presented a window of opportunity for Southern HSOs to adopt HR strategies by 2019. It remains unclear whether this opportunity was seized, by whom, and what the barriers were to doing so.

**Table 1:** Incidences and percent increases of overdose deaths in the Deep U.S. South, 2014 and 2019*

State	2014	2019	Percent Change
AL	723	768	6%
FL	2634	5268	100%
GA	1206	1408	18%
LA	777	1267	64%
MS	336	394	17%
NC	1358	2266	67%
SC	701	1127	61%
TN	1269	2089	65%
TX	2601	3177	22%

*Note.* From F.B. Ahmad, L.M. Rossen, and P. Sutton, *Provisional drug overdose death counts,* 2022, National Center for Health Statistics. (https://www.cdc.gov/nchs/nvss/vsrr/drug-overdose-data.htm#citation)

**Table 2:** Harm reduction-related legal conditions of Deep U.S. South in 2014 and 2019

Legal Condition	2014	2019
Law explicitly authorizes SSPs*	No Deep South state	FL, GA, LA, NC, TN
Does not have any paraphernalia law*	No data	None
Law does not prohibit simple possession of paraphernalia or syringes*	No data	None
Paraphernalia law provides immunity for persons who disclose possession of syringes to police officers prior to search	No data	NC, TN
Law exempts residue in used syringe from crime of drug possession*	No data	NC, TN
Law protecting naloxone distribution**	NC	AL, FL, GA, LA, NC, MS, SC, TN, TX
Laws protecting those who call emergency services for an overdose from drug law prosecution (“Good Samaritan” law)**	FL, NC	AL, FL, GA, LA, MS, NC, SC, TN

*Note.* The data for legal conditions demarcated with one asterisk (*) are from *State laws governing syringe services programs and participant syringe possession, 2014-2019*, by M. Fernández-Viña, N.E. Prood, A. Herpolsheimer, J. Waimberg, and S. Burris, 2020, (10.1177/0033354920921817). The data for legal conditions demarcated with two asterisks (**) are from *Naloxone Overdose Prevention Laws,* by Center for Public Health Law Research, 2017, (https://www.pdaps.org/datasets/laws-regulating-administration-of-naloxone-1501695139)

## Study aim two: survey of HR implementation among southern HSOs

Little is known about the extent to which Southern HSOs implement HR. Relatively low rates of opioid misuse within the communities served by HSOs in the early opioid epidemic, as well as lower rates of opioid overdose in the Deep South in general combined with policy hostility toward HR may have posed a formidable barrier to HR implementation prior to 2014. However, increased rates of overdose and HR-related policy changes between 2014 and 2019 may have spurred Southern HSOs to implement HR. A survey we conducted with HSOs in the Deep South in late 2018/early 2019 provides some insight into HR implementation among HSOs at this time*.*

### Methods

The authors are a part of a research team that administered a two-part survey to community-based organizations in the South that serve people living with HIV (PLWHA). The first survey collected information about existing HSOs, such as organizational characteristics (i.e., organization type, number of clients served each year, staff size) and services provided (i.e., HIV/STI, mental health care, substance abuse services), as well as organizational training history and needs. Upon completion of the first survey, respondents were invited to complete the second survey which collected information about perceived adequacy of services and organizational implementation of HR and trauma-informed care approaches. HR questions in the second survey asked about implementation of both specific HR services, as well as a broader HR-centered approach to care. To measure a HR-centered approach to care, survey respondents were asked to self-report whether or not 1) their organizational policies reflect a harm reduction orientation, 2) their organizational documents reflect an HR orientation and 3) their organization provided specific HR services. Examples of HR organizational policies and HR community-facing organizational documents were provided. This research was reviewed and approved by the University of Houston Institutional Review Board and survey respondents provided informed consent.

### Study sample and characteristics

Researchers utilized two databases to identify participants: 1) the National Prevention Information Network (NPIN), a database of HSOs, and 2) the U.S. Substance Abuse and Mental Health Services Association (SAMHSA), a database of behavioral health care providers. Organizations listed in the SAMHSA database must have indicated serving people living with HIV/AIDS to be eligible to participate in the survey. This manuscript analyzes data from 207 organizations who completed both surveys.

Organizations (*n* = 207) represented all nine states in the Deep South with 22% from Florida, 17% from Georgia, 15% from Texas, 10% from North Carolina, 10% from South Carolina, 7% from Louisiana, 6% from Alabama, 4% from Mississippi and 1% from multiple states. Organizations ranged from zero to 1607 full-time staff (*x̄*=67.3, SD = 183.7) and provided HIV services from less than 1 year to 50 years^1^ (*x̄*=19.3 years, SD = 11.7). The number of clients that these organizations served varied widely, ranging between one client and 1.2 million clients per year (*x̄*=14,806, SD = 95,238), with an average of 3637 PLWHA per year (SD = 36,907). Two extreme high volume outliers that represent large multi-site hospital systems are included in this data. The median number of clients served each year was 2000, and the median number of PLWHA served each year was 155. The majority of organizations surveyed provided substance use screenings (61%) and service referrals (71%). Half (50%) of organizations provided some form of substance misuse services. Seventy-six percent (76%) of organizations thought that existing substance misuse services were insufficient in meeting community needs.

### Data analysis

The research team performed frequency distributions on relevant survey questions.

### Results

Half of organizations surveyed reported that their policies reflected a HR orientation, and 58% reported that their organizational documentation reflected a HR orientation. Only 26% of organizations reported providing specific HR services. Specifically, 4% reported providing syringe service programming, 13% provided overdose reversal kits, 7% provided overdose reversal training, and 3% offered wound care training. Fourteen organizations (7%) reported implementing HR in “other” ways. However, some of the write-in responses for “other” HR approaches included things that may or may not be implemented in keeping with HR, such as substance use screening interventions (i.e., SBIRT) and abstinence-based group interventions (i.e., Alcoholics Anonymous, Narcotics Anonymous). Thirty-four organizations (16%) stated they did not implement HR.

Despite reported low rates of utilizing HR approaches, organizations indicated a strong interest. Of the 16% of organizations who reported that their organization does not utilize a HR approach, 74% indicated they would like to. Similarly, while only 36% of organizations had ever completed an organization-wide training in HR, 84% indicated interest in receiving such training.

Most organizations (55%) reported lack of funding as a barrier to utilizing HR approaches, as well as lack of expertise/knowledge (37%), staffing/capacity issues (36%), and the political climate (21%). Additional details are summarized in Table 3.

**Table 3:** Descriptive Statistics of HSOs, *n* = 207

	Mean	SD	Range
Organizational Characteristics			
Number of Staff	67.3	183.7	0-1607
Years providing HIV services (*n* = 165)	19.3	11.7	0-50
Number of clients (*n* = 164)^a^	14,806	95,238	1-1,200,000
Number of people provided substance use Services (*n* = 92)	825	4327	1-40,000
Number of clients living with HIV served in a year (*n* = 163)^a^	3637	36,907	1-475,100
	**N**	**%**	
States			
Alabama	13	6	
Florida	47	22	
Georgia	36	17	
Louisiana	16	7	
Mississippi	8	4	
North Carolina	21	10	
South Carolina	20	10	
Tennessee	12	6	
Texas	32	15	
Multiple States	2	1	
Substance Use Services			
Screening	126	61	
Referrals	146	71	
Treatment	103	50	
Current services are sufficient in meeting community needs	128	76	
Harm Reduction (HR) Implementation			
Organizational policies reflect a HR orientation	98	47	
Organization documents reflect HR orientation	120	58	
Organization provides specific HR services	53	26	
Syringe access/needle exchange	10	5	
Overdose reversal kit access	27	13	
Overdose reversal training	15	7	
Wound care training	6	3	
Safe injection sites	0	0	
HR education programming	99	48	
Organization does not take a HR approach	34	16	
Organization has participated in a HR training	75	36	
Barriers to Implementing HR			
Capacity/staffing	75	36	
Expertise/knowledge	76	37	
Funding	113	55	
Politics	44	21	

^a^Two extreme outliers that represent large multi-site hospital systems are included in this data. The median number of clients served each year was 2000, and the median number of PLWHA served each year was 155

## Study aim three: analysis of the impact of political and social contexts on HR implementation in the south

### Methods

Drawing from the analysis of the HR implementation context in the South presented in aim one, we were interested in whether differing contexts within the South impacted HSO implementation of HR.

### Variables

#### Independent variables

We constructed two independent variables to reflect variations in the HR context within the Deep South at the time of the survey. The first reflected states that saw the biggest percentage increases in drug-related mortality from 2014 to 2019 (FL, LA, NC, SC, TN) and enabled us to see if acuteness of need predicted HR implementation. We constructed a binary variable separating states that had a 50% or greater increase in overdose between 2014 and 2019 (FL, LA, NC, SC, TN) and those with less than 50%. The difference between these groups was stark; the next highest percent increase less than 50% was 22%. The second independent variable categorized states that, as of 2019, had at least some explicit legal protections for SSPs (FL, GA, LA, NC, TN). This enabled us to see if the policy environment influenced HR implementation. Though our survey was collected late 2018/early 2019, our experience on the ground indicated that states where SSPs were legally protected by 2019, but not before (i.e., FL and GA), were already operating in a policy environment generally more open to HR in late 2018 than states that did not have such legal protections by 2019 (AL, MS, SC, TX).

#### Dependent variables

We used four dependent variables to measure HR implementation: 1) whether or not HR was reflected in community-facing organizational documents, 2) whether or not HR was reflected in organizational policies, 3) whether the organization provided specific HR services (i.e., syringe, overdose reversal, wound care), and 4) whether the organization received training in HR.

### Data analysis

We constructed binomial logistic regressions to determine whether increased overdose rates and/or the policy environment were predictive of HSO implementation of HR. Analyses were adjusted for the number of years HSOs provided HIV services and number of staff (as a proxy for organization size). These covariates were selected to isolate and control for the effects of drug-related mortality rates and explicit legal protections for SSPs. Stata version 17 was used to conduct study analyses [57]. Statistical significance level (*p* value) was set at 0.05. We assessed data to be missing at random and utilized multiple imputation (10 imputations) to account for missing data since listwise deletion accounted for more than 10% of data [58].

### Results

The first set of models used increased state mortality rate as a predictor of HSO implementation of HR as demonstrated by integration in organizational documents, policies, services, and receipt of HR training. No models using increased mortality rate as an independent variable were statistically significant at the *p* < 0.05 level. However, the odds that organizations in states with higher increases in mortality included HR in their organizational documentation was notably higher than those with lower increases in mortality, with most of the 95% confidence interval above 1.0 (OR = 1.66, *p* = .17). The second set of models used HR-friendly policy context as a predictor of these same indicators of HSO implementation of HR. Organizations in states that had explicit legal protections for SSPs were more than twice as likely as those without such protections to have received training in HR (OR = 2.31, *p* = 0.02). Additionally, organizations in states with SSP protections were twice as likely to provide specific HR services than states without (OR = 2.28, *p* = 0.05). However, even among states with legal SSP protections, only about 32% of HSOs surveyed provided specific HR services (compared to 15% without legal protections). States with SSP protections were nor more or less likely than those without to include HR in their organizational policies or documents. Analysis results are summarized in Tables 4 and 5.

**Table 4:** Logistic regression analysis assessing associations between organizations in states with increased mortality rates^a^ 2014-2019 and harm reduction implementation among HSOs in the South

Dependent Variable	O.R. (95% C.I.)
HR in org docs	1.66 (0.82, 3.33)
HR in org policies	0.63 (0.32, 1.24)
HR services	0.66 (0.31, 1.37)
HR training	1.11 (0.56, 2.20)

^a^Controlling for number of years serving people living with HIV and number of full-time staff

**Table 5:** Logistic regression analysis assessing associations between organizations in states with legal SSP protections^a^ and harm reduction implementation among HSOs in the South

*Dependent Variable*	*O.R. (95% C.I.)*
HR in org docs	1.30 (0.62, 2.71)
HR in org policies	0.96 (0.48, 1.91)
HR services	2.31 (1.11, 4.77)
HR training	2.28 (1.10, 4.73)

^a^Controlling for number of years serving people living with HIV and number of full-time staff.

## Discussion

This study highlights how regions within high-income countries can face unique barriers to healthcare and therefore require a unique understanding of implementation context. Our analysis of the dynamics of the opioid epidemic in the U.S. South reveals a rapidly changing implementation context in which increased need meets increased political opportunity. Southern HSOs are in a moment where these landscape changes are being met with investments in organizational infrastructure, thus creating an opportunity for HSOs to meet HR needs in their communities.

Survey data indicates that there may be a lag between this opportunity and Southern HSOs’ ability to meet the moment. To our knowledge, this manuscript presents data from the largest survey of HSOs in the South that includes data related to HR. Findings reveal low rates of HSO provision of specific HR services or codifying HR approaches in organizational policies or community-facing documentation. Despite the context of increased deaths related to opioid use from 2014 to 2019 and high reported rates of providing substance misuse screenings, referrals and services, fewer than half of the organizations surveyed had ever completed HR training at the time of survey in 2019. This overall lack of HR adoption, however, does not indicate a lack of interest or recognition of the importance of HR among HSOs. Most organizations who did not implement any HR strategies were interested in doing so, and the majority of organizations reported wanting to receive HR training. Lack of funding to support HR was the most frequently cited barrier to implementation.

In the third study aim, we considered different contexts within the South that may have impacted HR adoption. The first context was whether a state had legal protections for SSPs in place by 2019. We used this as a proxy for a policy context more open to HR strategies. Our data showed that states with a more open policy context were significantly more likely to have received an organization-wide training on HR, indicating that this policy context may facilitate access to HR-related capacity building efforts. These states were also significantly more likely to provide HR services. However, only 32% of HSOs located in states with SSP protections provided specific HR services, though this was higher than the 16% of states without such protections. Policy environment did not predict broader implementation of a HR approach, possibly indicating an emphasis on HR services to the exclusion of HR as a person-centered organizational approach to care.

The second context we considered was that of drug-related mortality trends. Despite the increased urgency of HR needs, states with the greatest increase in drug mortality from 2014 to 2019 were no more likely than other Southern states to provide HR services, use HR organizational approaches, or receive HR training. This finding comports with research identifying a large percentage of counties with high prevalence of overdose that did not have any overdose education or naloxone distribution across the U.S. [30] On-the-ground need may influence HR legislation, as we saw in the policy changes from 2014 to 2019 as overdose rates in the South rose. However, increased need alone does not necessarily result in resources necessary for HR implementation. It may also indicate that other, non-HIV centered grassroots organizations may have mobilized earlier in the opioid epidemic in these states to provide HR-related services . However, an epidemic requires mobilization of all available resources and HSOs may be an underutilized resource in these hard-hit areas.

### Limitations

Our findings should be interpreted with limitations in mind. Firstly, our survey was only completed by HIV service organizations in the South and therefore does not include, nor account for, non-HIV related HR organizations that arose in response to the opioid epidemic in the South. The presence of these organizations may have impacted the extent to which HSO’s provide HR services. For example, HSO may refer clients to HR organizations for specific services to avoid service redundancy and respect HR-specific organizations’ ties to the community. The presence of HR organizations may also influence HSO adoption of HR organizational approaches by shifting local service provider attitudes to be more favorable or normalized to HR. Future research could examine these organizational interrelationships. Additionally, our survey relies on organizations self-reporting their implementation of HR. This self-report may be particularly unreliable for organizations that have not completed HR training and therefore may misunderstand core principles of HR. Research using non self-report data (i.e. organizational document review, ‘secret shopper’ studies) should be considered to further strengthen the evidence on HR organizational implementation.

## Implications

The U.S. federal administration has recently invested $30 million into addressing the opioid epidemic, which has only intensified during the COVID-19 pandemic [59, 60]. HSOs operate at the crossroads of two devastating epidemics and could be a powerful site of HR services. HSOs could also address issues with medical mistrust, client retention, and stigma by adopting a HR-oriented organizational approach. The low rates of HR training reported by HSOs in our sample indicate an important starting place, however training is just a start. Our previous research highlights the importance of in-depth training combined with coaching and implementation support to achieve the depth of organizational change necessary to support an holistic HR approach and integrated HR services [27]. As outer factor implementation determinants change and implementation opportunity opens up, funders of capacity building efforts to support HR must recognize the time and resources needed to affect meaningful change within organizations. As we have seen, legalization of high profile services such as SSPs is not enough to spur implementation of HR strategies and approaches, though lack of such legal protections may have a chilling effect on all HR activities within HSOs. We argue that a portion of the federal funding committed to this issue should go to this in-depth capacity building of Southern HSOs, particularly those that are Black and Latinx led. The intensifying opioid epidemic in the South is negatively impacting the same Black and Latinx communities that have been disproportionately impacted by the HIV epidemic due to structural drivers of health rooted in systematic racism. Black and Latinx-led Southern HSOs are uniquely suited to meet community needs through addressing the complexities of historic and current racialized trauma in the South, yet have traditionally been underfunded [60, 61]. The HR movement, in general, has been criticized for excluding the voices of Black and Latinx harm reductionists and insufficiently addressing structural racism in the context of HR, though this is beginning to change through the advocacy of Black and Latinx activists and allies [56, 62, 63].

Indeed, the shifting HR policy environment in the South has been realized by the sustained, committed work of HR activists. SSPs legislation change was hard-won and advocates continue to push the boundaries of HR services including recent work on supervised consumption sites as well as other HIV-related HR issues (e.g., sex work, HIV decriminalization) [64, 65]. However, legality of services does not necessarily ensure smooth implementation. Continued advocacy must center the ways in which people who use drugs may be legally harassed when trying to access HR services and HSOs must navigate byzantine restrictions on funding use, approvals and requirements to provide HR services [54]. Finally, policy advocacy must be met with broader social advocacy that reduces stigma around drug use and HIV that is at the root of HR hesitancy among Southern HSO leadership and staff . Grassroots organizations in the South can be a model for doing this work over the long term [62].

## Data Availability

The datasets used and/or analyzed during the current study are available from the corresponding author on reasonable request.

## Footnotes

1 Six organizations in the sample stated that they provided HIV service for longer than HIV had been present in the U.S. at the time of the survey (37 years). We interpret this to mean that these organizations mistakenly answered with total years providing services rather than just HIV services.
